# Rapid ESBL-Carba Combo PN test for simultaneous detection of ESBL and carbapenemase activity in Enterobacterales

**DOI:** 10.1128/spectrum.01123-26

**Published:** 2026-09-02

**Authors:** Soraya Herrera-Espejo, Jacqueline Findlay, Maxime Bouvier, Clément Viguier, Maurice Klahre, Nicolas Bodart, Patrice Nordmann

**Affiliations:** 1 Medical and Molecular Microbiology, Faculty of Science and Medicine, University of Fribourg 98839, Fribourg, Switzerland; 2 D-Tek SA, Mons, Belgium; 3 Swiss National Reference Center for Emerging Antibiotic Resistance (NARA), University of Fribourg 27211, Fribourg, Switzerland; Universita degli Studi dell'Insubria, Varese, Italy

**Keywords:** Enterobacterales, extended-spectrum β-lactamases, carbapenemase, rapid phenotypic detection test, chromogenic assay

## Abstract

A chromogenic biochemical assay (ESBL-Carba Combo PN test) was developed for the simultaneous detection of extended-spectrum β-lactamase (ESBL) and carbapenemase activity in Enterobacterales, as well as for discrimination of clinically relevant carbapenemase classes. The assay is based on a novel chromogenic substrate, B-Lac, and a low-cost Tris-based lysis buffer, providing a unified workflow for the detection of ESBLs and carbapenemases. The test was evaluated using cultures of well-characterized clinical isolates with resistance mechanisms previously defined by whole-genome sequencing. Results were obtained after a mean preparation time of approximately 3 min per isolate and were read after 20 min and 1 h of incubation. For ESBL detection (*n* = 67), sensitivity was 93.8% and 97.8% after 20 min and 1 h of incubation, respectively, with a specificity of 100%. Carbapenemase activity (*n* = 92) was detected with 100% sensitivity and specificity at both time points. Using the combined assay (*n* = 85), ESBL and/or carbapenemase activity was detected with 100% sensitivity and specificity. For Ambler classes A and B, 100% sensitivity and specificity were reached after 20 min of incubation. Regarding class D carbapenemases, 91.3% and 95.5% sensitivity after 20 min and 1 h of incubation, respectively, were observed, with 100% specificity at both times. The ESBL-Carba Combo PN test provides a simple, low-cost (<1 USD/sample), and functional method for the simultaneous detection of the major β-lactam resistance mechanisms in Enterobacterales and for carbapenemase class discrimination within one workflow, potentially supporting earlier optimization of antimicrobial therapy and antimicrobial stewardship in routine clinical microbiology laboratories.

**IMPORTANCE:**

Rapid identification of extended-spectrum β-lactamase (ESBL)- and carbapenemase-producing Enterobacterales (CPE) is essential to optimize antimicrobial therapy and implement timely infection control measures. Most currently available rapid phenotypic assays detect these resistance mechanisms separately, increasing hands-on time, workload, reagent consumption, and cost, particularly in isolates co-producing multiple β-lactamases. In addition, discrimination between carbapenemase classes, particularly class A and class D carbapenemases, remains challenging for most biochemical assays. This study describes a simple chromogenic assay that allows the simultaneous functional detection of both enzyme types using a single substrate and a low-cost lysis buffer. By incorporating relebactam as a selective class A inhibitor, the assay improves phenotypic discrimination between class A and class D carbapenemases while preserving reliable ESBL detection. The rapid ESBL-Carba Combo PN test provides 1 h of phenotypic information on the most clinically relevant β-lactam resistance mechanisms and represents a useful tool to guide early antibiotic treatment.

## INTRODUCTION

Rapid phenotypic detection of extended-spectrum β-lactamases (ESBLs) and carbapenemases in Enterobacterales remains a diagnostic priority because of the clinical impact of β-lactam resistance on patient outcomes (1, 2). Numerous studies have consistently demonstrated that mortality in severe infections increases in direct proportion to the delay in administration of an effective antimicrobial therapy (3). Therefore, early and accurate identification of antibiotic resistance mechanisms represents the basis of sepsis management and is strongly associated with improved survival.

However, currently available phenotypic assays target these resistance mechanisms separately, limiting diagnostic efficiency in isolates co-producing ESBLs and carbapenemases. Molecular assays allow sensitive detection of resistance genes but do not directly assess whether these determinants are expressed or functionally active under the tested conditions. In addition, they do not include all types of ESBL or carbapenemase targets. In contrast, phenotypic assays provide a direct readout of enzymatic activity, although they may be limited in detecting low-level or weakly expressed mechanisms.

The current prevalence of Enterobacterales co-producing ESBLs and carbapenemases complicates phenotypic detection strategies and highlights the need for integrated assays capable of identifying both resistance mechanisms within a single workflow (4). Several biochemical assays based on chromogenic or colorimetric substrates have been developed to detect β-lactamase activity directly from bacterial colonies, including tests targeting carbapenemases such as Rapid Carba NP test (5), NitroSpeed-Carba NP (6), or ESBLs like Rapid ESBL NP test (7). These assays rely on the hydrolysis of β-lactam substrates and the subsequent pH-dependent color change of a chromogenic indicator, allowing results to be obtained within minutes to hours. Although effective, they usually require different substrates and separate workflows for ESBL and carbapenemase detection (6).

In addition, accurate identification of class D carbapenemases, such as OXA-48-like enzymes, remains challenging for most biochemical assays and represents a well-recognized limitation of current phenotypic methods. This difficulty has been attributed to the weak and variable hydrolytic activity of OXA-48-like enzymes against vaborbactam (8, 9) and their atypical inhibition patterns reported for these biochemical methods (10, 11). These limitations highlight the need for optimized inhibitor strategies to enhance class D discrimination while maintaining a simple and rapid phenotypic workflow. In this study, a chromogenic biochemical assay based on a novel β-lactam chromogenic substrate (B-Lac; D-Tek, Belgium) was developed to enable the combined detection of ESBL and carbapenemase activity in Enterobacterales within a single, unified workflow.

## MATERIALS AND METHODS

### Bacterial isolates

A collection of 160 clinical Enterobacterales isolates from different sources (including sputum, urinary, blood culture, or skin), obtained from French and Swiss reference collections and with well-characterized β-lactam resistance mechanisms, was selected to evaluate the performance of the assay. Isolates included ESBL producers, carbapenemase-producing Enterobacterales (CPE; Ambler classes A, B, and D), strains co-producing ESBLs and carbapenemases, and β-lactamase-negative control strains. All resistance mechanisms had been previously characterized by molecular and/or phenotypic reference methods. Whole-genome sequencing served as the reference standard for resistance mechanism assignment. Readers performing the assay were blinded to the genotypic results.

### ESBL detection

For the ESBL detection assay, three 1.5-mL tubes were prepared (tubes A, B, and C). Then 100 and 50 µL of distilled water were added to tubes A and B, respectively. Then, 50 µL of lysis buffer (Tris-HCl 20 mmol/L, pH 7.5, 0.1% Triton X-100, 0.1 mM ZnSO₄) were dispensed into three 1.5-mL tubes. Tube A contained no antibiotic; tube B contained cefotaxime at a final concentration of 3 mg/L; and tube C contained cefotaxime (CTX; 3 mg/L) plus tazobactam at a final concentration of 4 mg/L. One full calibrated 1-µL loop of bacterial colonies was resuspended. After resuspension, 50 µL of B-Lac chromogenic reagent (250 µM, D-Tek, Belgium) was added to each tube. Tubes were vortexed briefly and incubated at 37°C. Results were read after 20 min and again after 1 h of incubation (Fig. S1A). Interpretation criteria were as follows: (i) negative result (no ESBL): the three tubes remained yellow; (ii) positive ESBL result: tubes A and B turned purple (hydrolysis of B-Lac), while tube C remained yellow; and (iii) undetermined result: all tubes turned purple, corresponding to possible AmpC production, ESBL + AmpC coproduction, or carbapenemase production with or without additional ESBL and/or AmpC activity (Fig. S1B).

### Carbapenemase detection

For carbapenemase detection and classification, five 1.5-mL tubes were prepared, 100 and 50 µL of distilled water were added to tubes 1 and 2, respectively. One calibrated 1-µL loop of colonies was resuspended: in tubes 1 and 2 containing 50 µL of the homemade lysis buffer; tube 3 containing 50 µL of Tris-HCl +50 µL of dipicolinic acid (DPA, 50 mM; Sigma-Aldrich); tube 4 containing 50 µL of Tris-HCl +50 µL of avibactam sodium hydrate (0.08 mM; MedChemExpress); and tube 5 containing 50 µL of Tris-HCl +50 µL of relebactam (100 µg/mL; MedChemExpress). The tubes were vortexed vigorously for 30 s until complete resuspension. From tubes 2 to 5, 50 µL of ertapenem sodium (80 µg/mL; Sigma-Aldrich) was added. Finally, 50 µL of B-Lac chromogenic reagent (D-Tek, Mons, Belgium) was added to each tube (Fig. S2A) before briefly vortexing and incubation at 37°C. Results were read after 20 min and again after 1 h of incubation. Interpretation criteria were (i) negative result (no ESBL/no carbapenemase): all tubes remained yellow; (ii) positive carbapenemase production: tubes 1 and 2 turned purple. Additionally, carbapenemase classification could be interpreted: (iii) class A carbapenemase: tubes 1, 2, and 3 turned purple, and tubes 4 and 5 remained unchanged; (iv) class B carbapenemase: tubes 1, 2, 4, and 5 hydrolyzed the B-Lac, turning the color to purple, but no color change in tube 3; (v) class D carbapenemase: tubes 1, 2, 3, and 5 turned purple, and tube 4 remained yellow (Fig. S2B).

### Rapid ESBL-Carba Combo PN test

**Fig 1:**
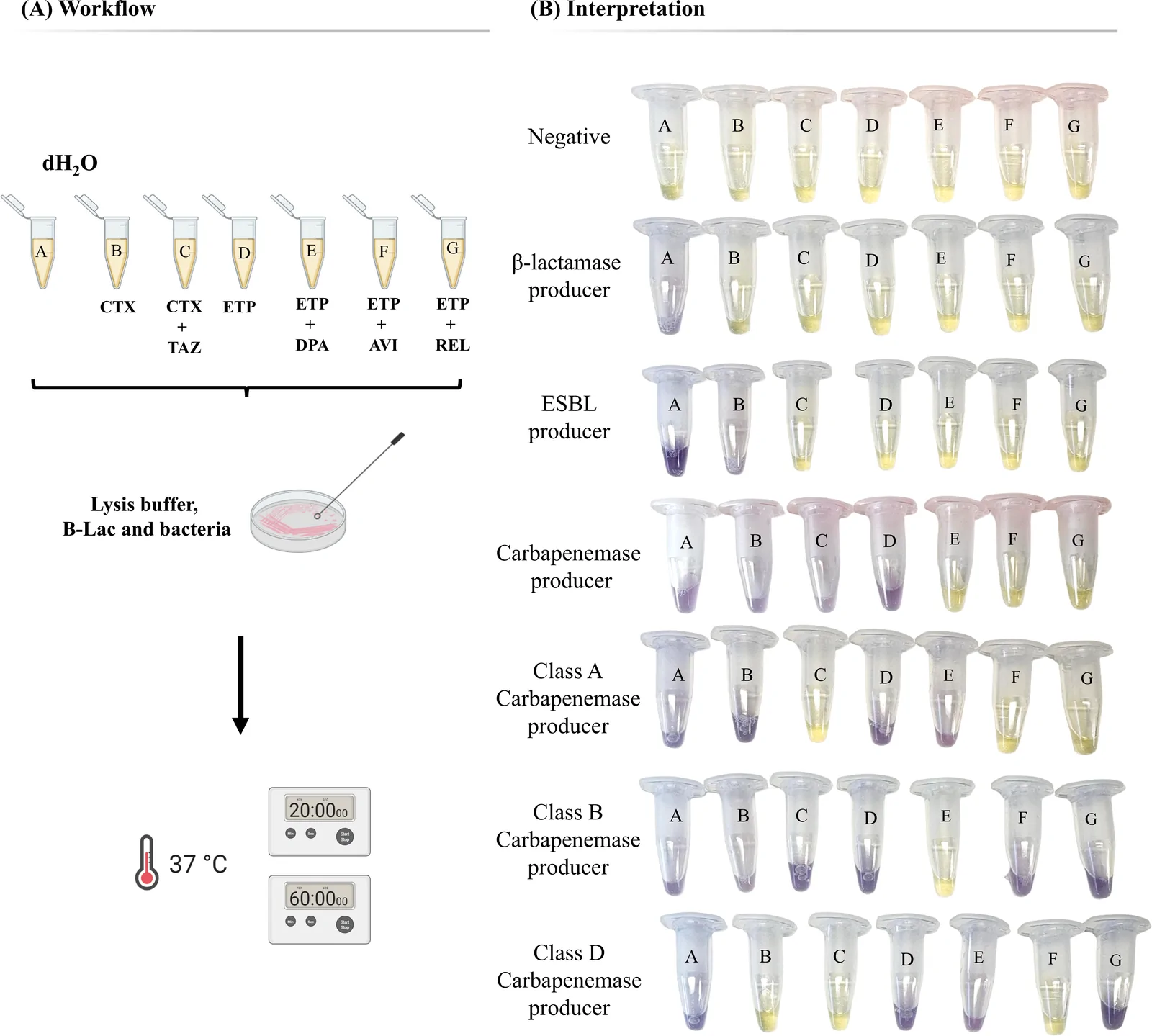
Schematic representation (**A**) and interpretation (**B**) for Rapid ESBL-Carba Combo PN Test. Tubes A, B, and C: rapid detection of ESBL; tubes D, E, F, and G: detection and classification of carbapenemases. _d_H_2_O: distilled water; CTX: cefotaxime; TAZ: tazobactam; ETP: ertapenem; DPA: dipicolinic acid; AVI: avibactam; REL: relebactam.

The combo PN test combined both ESBL and carbapenemase detection rapid tests using a single chromogenic substrate (B-Lac) and the same Tris-HCl/Triton X-100/ZnSO₄ lysis buffer for all reactions. For each isolate, a total of seven tubes were prepared: three tubes (A, B, and C) corresponding to the ESBL detection assay and four tubes (D, E, F, and G) corresponding to the carbapenemase detection and classification assay from tubes 2 to 5. All reactions were performed in parallel from fresh bacterial colonies (Fig. 1).

### Statistical analysis

Sensitivity and specificity were calculated using whole-genome sequencing as the reference standard. For the Combo PN test, a true positive result was defined as the correct detection of ESBL and/or carbapenemase activity in a given isolate. Performance was also evaluated separately for ESBL detection, carbapenemase detection, and assignment for Ambler class carbapenemase (A, B, and D). Exact 95% confidence intervals (CIs) for proportions were calculated using the Clopper–Pearson method. Results were analyzed at 20 min and after 1 h of incubation.

## RESULTS

The species distribution of the 160 total isolates was as follows: 76 (46.6%) *Escherichia coli*, 69 (42.3%) *Klebsiella pneumoniae*, 13 (8.0%) *Enterobacter cloacae*, 1 (0.6%) *Morganella morganii*, and 1 (0.6%) *Proteus mirabilis*. All isolates had been previously fully characterized by whole-genome sequencing.

### Rapid ESBL detection

The ESBL assay was evaluated using a collection of 67 Enterobacterales isolates producing a wide diversity of ESBLs. Importantly, the assay detected ESBL activity in isolates co-producing carbapenemases, including KPC, NDM, and OXA-48-like enzymes. After 20 min of incubation, ESBL activity was correctly identified in 42 of 45 ESBL-producing isolates, corresponding to a sensitivity of 93.8% (with a 95% confidence interval [CI_95_] from 81.8% to 98.4%) and a specificity of 100% (CI_95_: 84.6%–100%), with no false-positive (FP) results and three false-negative (FN) results. After 1 h of incubation, the number of FN results decreased to one, yielding a sensitivity of 97.8% (CI_95_: 88.5%–99.9%) and a specificity of 100% (CI_95_: 84.6%–100%) with no FP results (Table 1; Table S1).

**TABLE 1:** Analysis of the sensitivity, specificity, precision, and accuracy in the ESBL or carbapenemase activity using Rapid ESBL-Carba Combo PN Test

Test (N)	Incubation time	True positives	True negatives	False negatives	False positives	Sensitivity		Specificity		Accuracy (%)	Precision (%)	ME (%)	VME (%)
						%	95% CI	%	95% CI				
ESBL alone (*n* = 67)	20 min	45	22	3	0	93.8	81.8–98.4	100.0	84.6–100	95.7	100	**0**	6.3
	1 h	45	22	1	0	97.8	88.5–99.9	100.0	84.6–100	98.5	100	**0**	**2.2**
Carbapenemase alone (*n* = 92)	20 min	75	17	0	0	100.0	95.2–100	100.0	80.5–100	100	100	**0**	**0**
	1 h	75	17	0	0	100.0	95.2–100	100.0	80.5–100	100	100	**0**	**0**
Rapid ESBL + Carba Combo PN test (*n* = 85)	20 min	78^*a*^	7	0	0	100.0	95.4–100	100.0	59.0–100	100	100	**0**	**0**
	1 h	78^*a*^	7	0	0	100.0	95.4–100	100.0	59.0–100	100	100	**0**	**0**
- Class A	20 min	11	74	0	0	100.0	74.1–100	100.0	95.1–100	100	100	**0**	**0**
	1 h	11	74	0	0	100.0	74.1–100	100.0	95.1–100	100	100	**0**	**0**
- Class B	20 min	28	57	0	0	100.0	87.9–100	100.0	93.7–100	100	100	**0**	**0**
	1 h	28	57	0	0	100.0	87.9–100	100.0	93.7–100	100	100	**0**	**0**
- Class D	20 min	21^*a*^	64	2	0	91.3	73.2–98.7	100.0	94.3–100	97.7	100	**0**	8.7
	1 h	21^*a*^	64	1	0	95.5	78.2–99.8	100.0	94.3–100	98.8	100	**0**	4.5

^*a*^ Undeterminate results (*n* = 7), represented in the Table 2 with “U,” were excluded for the calculation of sensitivity, specificity, accuracy and precision; Boldface indicates parameters or values meeting the CLSI standards; MEs, major errors; VMEs, very major errors.

### Rapid carbapenemase detection

Carbapenemase detection was assessed using a collection of 92 Enterobacterales isolates producing Ambler classes A (KPC), B (NDM, VIM, IMP), and D (OXA-48-like) carbapenemases, together with non-carbapenemase-producing controls. At both 20 min and 1 h of incubation, carbapenemase activity was detected in all carbapenemase-producing isolates, corresponding to a sensitivity and specificity of 100% with a CI_95_: 95.2%–100% and CI_95_: 80.5%–100%, respectively, with no FP results. Carbapenemase class assignment based on inhibitor profiles correctly identified all class A and B using avibactam, DPA, respectively. Regarding class D enzymes, two isolates were misclassified at 20 min of incubation, and one after 1 h of incubation (Table 1; Table S2).

### Rapid ESBL-Carba Combo PN test

The Combo PN test detection was evaluated using 85 Enterobacterales isolates (Table 2) expressing diverse resistance mechanisms, including isolates co-producing ESBLs and carbapenemases. At both 20 min and 1 hour of incubation, the Combo PN test yielded a sensitivity and specificity of 100% with a CI_95_: 95.4%–100% and CI_95_: 59.0%–100%, respectively. Regarding carbapenemase class discrimination, the Combo PN test correctly identified all class A and B carbapenemases at both time points, 100% sensitivity and specificity. At 20 min, two FN were found, corresponding to a sensitivity of 91.3% (CI_95_: 73.2%–98.7%) and a specificity of 100% (CI_95_: 94.3%–100%) for class D carbapenemases. After 1 h of incubation, only one isolate remained misclassified, yielding a sensitivity of 95.5% (CI_95_: 78.2%–99.8%). Seven isolates producing OXA-48-like co-producing ESBL showed indeterminate results due to the lack of tazobactam inhibition. These seven isolates were not considered for the calculation of the performance metrics of this test (Table 1).

**TABLE 2:** Results of the combination test detecting ESBL and carbapenemase on Enterobacterales isolates after 20 min and 1 h of incubation^*a*^

ID	Strain	Class	Genetic characterization			20 min of incubation							Interpre-tation	1 hour of incubation							Interpre-tation
			Carbapenemases	ESBLs	Acquired βlactamases	-	+ CTX	+CTX/TAZ	+ ETP	+ETP/DPA	+ETP/AVI	+ETP/REL		−	+ CTX	+CFX/TAZ	+ ETP	+ETP/DPA	+ETP/AVI	+ETP/REL	
1	*E. coli*	*A*	KPC-2	CTX-M-15	SHV-1	+	+	−	+	+	−	−	**✔**	+	+	−	+	+	−	−	**✔**
2	*E. cloacae*		KPC-2	–	OXA-1	+	+	−	+	+	−	−	**✔**	+	+	−	+	+	−	−	**✔**
3	*K. pneumoniae*		KPC-2	CTX-M-15	TEM-1, OXA-1	+	+	−	+	+	−	−	**✔**	+	+	−	+	+	−	−	**✔**
4	*K. pneumoniae*		KPC-2	–	TEM-1	+	+	−	+	+	−	−	**✔**	+	+	−	+	+	−	−	**✔**
5	*K. pneumoniae*		KPC-2	–	TEM-1	+	+	−	+	+	−	−	**✔**	+	+	−	+	+	−	−	**✔**
6	*K. pneumoniae*		KPC-2	CTX-M-15	TEM-1, OXA-1	+	+	−	+	+	−	−	**✔**	+	+	−	+	+	−	−	**✔**
7	*K. pneumoniae*		KPC-3	SHV-11	–	+	+	−	+	+	−	−	**✔**	+	+	−	+	+	−	−	**✔**
8	*K. pneumoniae*		KPC-3	–	TEM-1	+	+	−	+	+	−	−	**✔**	+	+	−	+	+	−	−	**✔**
9	*K. pneumoniae*		KPC-3	SHV-11	CMY-16	+	+	−	+	+	−	−	**✔**	+	+	−	+	+	−	−	**✔**
10	*K. pneumoniae*		KPC-3	CTX-M-15	OXA-1	+	+	−	+	+	−	−	**✔**	+	+	−	+	+	−	−	**✔**
11	*K. pneumoniae*		KPC-3	–	TEM-1, OXA-9	+	+	−	+	+	−	−	**✔**	+	+	−	+	+	−	−	**✔**
12	*E. coli*	*B*	NDM-1	–	–	+	+	+	+	−	+	+	**✔**	+	+	+	+	−	+	+	**✔**
13	*E. coli*		NDM-1	–	OXA-1, CMY-4	+	+	+	+	−	+	+	**✔**	+	+	+	+	−	+	+	**✔**
14	*K. pneumoniae*		NDM-1	–	TEM-1	+	+	+	+	−	+	+	**✔**	+	+	+	+	−	+	+	**✔**
15	*K. pneumoniae*		NDM-1	CTX-M-15	OXA-1, TEM-1, OXA-9	+	+	+	+	−	+	+	**✔**	+	+	+	+	−	+	+	**✔**
16	*K. pneumoniae*		NDM-1	CTX-M-15	LAP-2	+	+	+	+	−	+	+	**✔**	+	+	+	+	−	+	+	**✔**
17	*K. pneumoniae*		NDM-1	CTX-M-15	LAP-2	+	+	+	+	−	+	+	**✔**	+	+	+	+	−	+	+	**✔**
18	*K. pneumoniae*		NDM-1	CTX-M-15	–	+	+	+	+	−	+	+	**✔**	+	+	+	+	−	+	+	**✔**
19	*E. coli*		NDM-1	–	OXA-10	+	+	+	+	−	+	+	**✔**	+	+	+	+	−	+	+	**✔**
20	*K. pneumoniae*		NDM-1	CTX-M-15	OXA-1, TEM-1, OXA-9	+	+	+	+	−	+	+	**✔**	+	+	+	+	−	+	+	**✔**
21	*K. pneumoniae*		NDM-1	CTX-M-15	TEM-1	+	+	+	+	−	+	+	**✔**	+	+	+	+	−	+	+	**✔**
22	*E. coli*		NDM-5	–	CMY-42	+	+	+	+	−	+	+	**✔**	+	+	+	+	−	+	+	**✔**
23	*E. coli*		NDM-5	CTX-M-15	CMY-42, TEM-1	+	+	+	+	−	+	+	**✔**	+	+	+	+	−	+	+	**✔**
24	*E. coli*		NDM-5	CTX-M-15	OXA-1	+	+	+	+	−	+	+	**✔**	+	+	+	+	−	+	+	**✔**
25	*K. pneumoniae*		NDM-5	–	–	+	+	+	+	−	+	+	**✔**	+	+	+	+	−	+	+	**✔**
26	*E. coli*		NDM-5	–	–	+	+	+	+	−	+	+	**✔**	+	+	+	+	−	+	+	**✔**
27	*E. coli*		NDM-5	–	–	+	+	+	+	−	+	+	**✔**	+	+	+	+	−	+	+	**✔**
28	*E. coli*		NDM-19	–	–	+	+	+	+	−	+	+	**✔**	+	+	+	+	−	+	+	**✔**
29	*K. pneumoniae*		VIM-1	SHV-5	–	+	+	+	+	−	+	+	**✔**	+	+	+	+	−	+	+	**✔**
30	*K. pneumoniae*		VIM-1	–	–	+	+	+	+	−	+	+	**✔**	+	+	+	+	−	+	+	**✔**
31	*K. pneumoniae*		VIM-4	–	–	+	+	+	+	−	+	+	**✔**	+	+	+	+	−	+	+	**✔**
32	*E. coli*		VIM-19	–	CMY-4	+	+	+	+	−	+	+	**✔**	+	+	+	+	−	+	+	**✔**
33	*K. pneumoniae*		VIM-19	CTX-M-3, TEM-1	CMY-4	+	+	+	+	−	+	+	**✔**	+	+	+	+	−	+	+	**✔**
34	*K. pneumoniae*		VIM-19	CTX-M-3	TEM-1, CMY-4	+	+	+	+	−	+	+	**✔**	+	+	+	+	−	+	+	**✔**
35	*K. pneumoniae*		IMP-1	TEM-15	–	+	+	+	+	−	+	+	**✔**	+	+	+	+	−	+	+	**✔**
36	*K. pneumoniae*		IMP-1	OXA-2, TEM-15	–	+	+	+	+	−	+	+	**✔**	+	+	+	+	−	+	+	**✔**
37	*K. pneumoniae*		IMP-2	TEM-15, OXA-2	–	+	+	+	+	−	+	+	**✔**	+	+	+	+	−	+	+	**✔**
38	*K. pneumoniae*		IMP-4	–	TEM-1	+	+	+	+	−	+	+	**✔**	+	+	+	+	−	+	+	**✔**
39	*K. pneumoniae*		IMP-8	–	LAP-2	+	+	+	+	−	+	+	**✔**	+	+	+	+	−	+	+	**✔**
40	*P. mirabilis*	*D*	OXA-23	–	–	+	−	−	+	+	−	+	**✔**	+	−	−	+	+	−	+	**✔**
41	*E. coli*		OXA-48	CTX-M-14	–	+	+	+	+	+	−	+	**U**	+	+	+	+	+	−	+	**U**
42	*E. coli*		OXA-48	CTX-M-24	–	+	+	+	+	+	−	+	**U**	+	+	+	+	+	−	+	**U**
43	*E. coli*		OXA-48	–	–	+	−	−	+	+	−	+	**✔**	+	−	−	+	+	−	+	**✔**
44	*K. pneumoniae*		OXA-48	CTX-M-15	TEM-1, OXA-1	+	+	+	+	+	−	+	**U**	+	+	+	+	+	−	+	**U**
45	*K. pneumoniae*		OXA-48	CTX-M-15	OXA-1	+	+	−	+	+	−	+	**✔**	+	+	−	+	+	−	+	**✔**
46	*E. coli*		OXA-181	CTX-M-27	–	+	+	−	+	+	−	+	**✔**	+	+	−	+	+	−	+	**✔**
47	*E. cloacae*		OXA-181	CTX-M-15	OXA-9	+	+	−	+	+	−	+	**✔**	+	+	−	+	+	−	+	**✔**
48	*K. pneumoniae*		OXA-181	CTX-M-15	OXA-1	+	+	−	+	+	−	+	**✔**	+	+	−	+	+	−	+	**✔**
49	*K. pneumoniae*		OXA-204	CTX-M-14, CTX-M-15	CMY-4, OXA-1, TEM-1	+	+	+	+	+	−	+	**✔**	+	+	+	+	+	−	+	**✔**
50	*E. coli*		OXA-204	CTX-M-15	OXA-1, CMY-4	+	+	−	+	+	−	-	**M**	+	+	−	+	+	−	+	**✔**
51	*K. pneumoniae*		OXA-232	CTX-M-15	OXA-1	+	+	+	+	+	−	+	**✔**	+	+	+	+	+	−	+	**✔**
52	*E. coli*		OXA-244	–	–	+	−	−	+	+	−	+	**✔**	+	-	−	+	+	−	+	**✔**
53	*E. coli*		OXA-244	–	–	+	−	−	+	+	−	+	**✔**	+	-	−	+	+	−	+	**✔**
54	*E. coli*		OXA-244	CTX-M-15	–	+	+	−	+	+	−	+	**✔**	+	+	−	+	+	−	+	**✔**
55	*E. coli*		OXA-244	CTX-M-14	TEM-1	+	+	+	+	+	−	−	**U / M**	+	+	+	+	+	−	−	**U / M**
56	*E. coli*		OXA-244	–	–	+	−	−	+	+	−	+	**✔**	+	−	−	+	+	−	+	**✔**
57	*E. coli*		OXA-244	CTX-M-27	–	+	+	+	+	+	−	+	**U**	+	+	+	+	+	−	+	**U**
58	*E. coli*		OXA-244	–	–	+	+	+	+	+	−	+	**U**	+	+	+	+	+	−	+	**U**
59	*E. coli*		OXA-244	CTX-M-14	TEM-1	+	+	+	+	+	−	+	**U**	+	+	+	+	+	−	+	**U**
60	*E. coli*		OXA-244	CTX-M-14	TEM-1	+	+	−	+	+	−	+	**✔**	+	+	−	+	+	−	+	**✔**
61	*K. pneumoniae*	*Double carbapenemases*	NDM-1, OXA-232	CTX-M-15	CMY-4, TEM-1	+	+	+	+	+	+	+	**✔**	+	+	+	+	+	+	+	**✔**
62	*E. coli*		NDM-5, OXA-244	–	–	+	+	+	+	+	+	+	**✔**	+	+	+	+	+	+	+	**✔**
63	*K. pneumoniae*	*Non-carbapenemase producers + ESBL producers*	–	CTX-M-3	TEM-1	+	+	−	−	−	−	−	**✔**	+	+	−	−	−	−	−	**✔**
64	*E. coli*		–	CTX-M-3	–	+	+	−	−	−	−	−	**✔**	+	+	−	−	−	−	−	**✔**
65	*E. coli*		–	CTX-M-3	–	+	+	−	−	−	−	−	**✔**	+	+	−	−	−	−	−	**✔**
66	*E. cloacae*		–	CTX-M-3	TEM-1	+	+	−	−	−	−	−	**✔**	+	+	−	−	−	−	−	**✔**
67	*K. pneumoniae*		–	CTX-M-15	–	+	+	−	−	−	−	−	**✔**	+	+	−	−	−	−	−	**✔**
68	*E. coli*		–	CTX-M-15	–	+	+	−	−	−	−	−	**✔**	+	+	−	−	−	−	−	**✔**
69	*K. pneumoniae*		–	CTX-M-15	–	+	+	−	−	−	−	−	**✔**	+	+	−	−	−	−	−	**✔**
70	*E. coli*		–	CTX-M-15	–	+	+	−	−	−	−	−	**✔**	+	+	−	−	−	−	−	**✔**
71	*K. pneumoniae*		–	CTX-M-15	TEM-1	+	+	−	−	−	−	−	**✔**	+	+	−	−	−	−	−	**✔**
72	*K. pneumoniae*		–	CTX-M-15	OXA-1, TEM-1	+	+	−	−	−	−	−	**✔**	+	+	−	−	−	−	−	**✔**
73	*E. cloacae*		–	CTX-M-15	OXA-1, TEM-1	+	+	−	−	−	−	−	**✔**	+	+	−	−	−	−	−	**✔**
74	*M. morganii*		–	CTX-M-15	DHA-1, OXA-1, TEM-1	+	+	−	−	−	−	−	**✔**	+	+	−	−	−	−	−	**✔**
75	*E. coli*		–	CTX-M-55	–	+	+	−	−	−	−	−	**✔**	+	+	−	−	−	−	−	**✔**
76	*K. pneumoniae*		–	CTX-M-33	OXA-1, TEM-1	+	+	−	−	−	−	−	**✔**	+	+	−	−	−	−	−	**✔**
77	*K. pneumoniae*		–	OXA-2	–	+	+	−	−	−	−	−	**✔**	+	+	−	−	−	−	−	**✔**
78	*E. cloacae*		–	TEM-3	–	+	+	−	−	−	−	−	**✔**	+	+	−	−	−	−	−	**✔**
79	*K. pneumoniae*	*Non-carbapenemase/ Non-ESBL producers*	–	–	–	+	−	−	−	−	−	−	**✔**	+	−	−	−	−	−	−	**✔**
80	*K. pneumoniae*		–	–	–	+	−	−	−	−	−	−	**✔**	+	−	−	−	−	−	−	**✔**
81	*K. pneumoniae*		–	–	–	+	−	−	−	−	−	−	**✔**	+	−	−	−	−	−	−	**✔**
82	*E. cloacae*		–	–	–	+	−	−	−	−	−	−	**✔**	+	−	−	−	−	−	−	**✔**
83	*E. coli*		–	–	–	−	−	−	−	−	−	−	**✔**	−	−	−	−	−	−	−	**✔**
84	*E. coli*		–	–	–	−	−	−	−	−	−	−	**✔**	−	−	−	−	−	−	−	**✔**
85	*E. coli*		*–*	*–*	–	−	−	−	−	−	−	−	**✔**	−	−	−	−	−	−	−	**✔**

^*a*^ +, hydrolisis (from yellow to purple color); −, absence of hydrolisis (no color change); ✔, expected results; M, misidentification of carbapenemase class; U, undetermined results.

## DISCUSSION

This study describes the development of a chromogenic biochemical assay allowing the combined detection of ESBLs and carbapenemases activities in Enterobacterales. The proposed approach integrates both detections into a single workflow using one chromogenic substrate and a common lysis buffer, thereby simplifying laboratory handling and reducing reagent cost. This combined workflow represents an improvement over currently available rapid biochemical tests, which generally require separate methods for ESBL and carbapenemase detection.

Beyond its diagnostic performance, the assay offers practical and economic advantages compared with previously described biochemical procedures. Conventional ESBL-NP and Carba-NP assays for examples rely on commercial lysis buffers such as B-PER (ThermoFisher Scientific, USA) (6, 12), which represent a higher cost (approximately 840 USD per 250 mL) per sample, whereas the present test uses an inexpensive Tris-HCl-based buffer. In addition, nitrocefin, the chromogenic substrate commonly used in the previous reported assays, is costly (approximately 1.5 USD per 100 mL) and chemically unstable, requiring daily preparation of fresh working solutions. By comparison, the B-Lac substrate is significantly cheaper (approximately 100 USD per 250 mL) and remains stable for up to 9 months when stored at 4°C. This improved stability reduces reagent waste and hands-on time, thereby enhancing suitability for routine laboratory implementation. All these features may be particularly advantageous for high-throughput laboratories and for resource-limited settings, and particularly relevant in light of recently described low-cost colorimetric assays for carbapenemase detection, ResaCPE test (13). Although this protocol requires prolonged incubation times of up to 3.5 h to yield interpretable results and is restricted to carbapenemase detection only. Conversely, the Combo PN test provides reliable functional results within 20 min, enables the simultaneous detection of both ESBL and carbapenemase activities, and relies on inexpensive and stable reagents.

The Combo PN test enables discrimination of ESBL activity through the use of cefotaxime, an extended-spectrum cephalosporin with high sensitivity to hydrolysis by most ESBLs, particularly CTX-M-type enzymes, which are highly prevalent in Enterobacterales. This makes cefotaxime a suitable substrate for detecting ESBL activity in a broad range of clinical isolates. Tazobactam was selected because of its activity against class A ESBLs. Compared with clavulanic acid, tazobactam offers greater stability under the assay conditions and reduced interference in chromogenic readouts, which improves the robustness and interpretability of the test.

Significantly, the ability of the Combo PN test to discriminate between carbapenemase classes has major therapeutic implications. Increasingly, treatment strategies for infections caused by carbapenemase-producing Enterobacterales rely on β-lactam/β-lactamase inhibitor (BL/BLI) combinations with class-dependent activity (14). For example, class A enzymes, such as KPC, are usually inhibited by avibactam, vaborbactam, and relebactam, making ceftazidime-avibactam or meropenem-vaborbactam or imipenem-relebactam effective options (15, 16). In contrast, metallo-β-lactamases (class B) are not inhibited by these compounds but remain susceptible to aztreonam, which is stable to MBL hydrolysis; however, aztreonam is often co-hydrolyzed by ESBLs or AmpC enzymes, requiring combination with avibactam to restore activity (17). One-hour functional classification may enable earlier selection of BL/BLI combinations for treating infections associated with MBL producers. Rapid identification of an MBL profile may support, for example, the early use of aztreonam–avibactam-based regimens. Moreover, in the near future, novel antibiotic combinations containing MBL inhibitors such as those containing xeruborbactam and taniborbactam may be marketed (18, 19).

Identification of OXA-48-like carbapenemases (class D) is also important since they may respond to ceftazidime-avibactam (9). Previous rapid biochemical assays enabling discrimination of class D carbapenemases, particularly OXA-48-like enzymes, remained unreliable (20–22). In contrast, the Combo PN test demonstrated improved performance for the discrimination of class D carbapenemases by incorporating relebactam as part of a differential inhibitor strategy. Although the sensitivity for class D detection did not reach 100%, the assay demonstrated a high identification rate for OXA-48-like producers, supporting its clinical usefulness. In Table 2, isolates labeled as “M” correspond to misclassified carbapenemase class results, whereas those labeled as “U” were considered undetermined. The false-negative results obtained for some of the OXA-204 and OXA-244 producers could be explained by weak carbapenemase activity and/or gene expression resulting in partial inhibition following relebactam addition (23, 24). Nevertheless, 82% (9/11) of OXA-204/OXA-244 producers were correctly identified.

On the other hand, isolates categorized as undetermined (“U”) corresponded to cases in which class D carbapenemases were correctly identified, but an unexpected lack of inhibition by tazobactam prevented full phenotypic interpretation within the ESBL detection component of the assay. This phenomenon, observed in five out of the seven discrepant isolates, may be explained by chromosomal AmpC overproduction, which can interfere with the tazobactam activity. This hypothesis was further supported by additional antimicrobial susceptibility testing using cloxacillin-containing plates (data not shown), confirming an AmpC hyperproduction phenotype in these isolates.

This improved discrimination achieved with the Combo PN test highlights the added value of the combined inhibitor strategy. In this context, a rapid phenotypic assay providing information on carbapenemase class may guide the rapid selection of the most appropriate targeted therapy, improve antimicrobial stewardship, and potentially reduce mortality associated with inappropriate empirical treatment. Although this proof of concept was established using bacterial cultures, future studies should evaluate whether the assay can be adapted for direct detection of ESBLs and carbapenemases in bloodstream infections. Positive blood cultures typically contain bacterial concentrations ranging from 10^7^ to 10^10^ CFU/mL, which should be sufficient to perform the assay after simple sample processing. However, this approach would require further validation, including appropriate ethical approval for the use of human-derived specimens.

The main limitations of this study are (i) the limited number of certain carbapenemase subtypes, which precluded robust statistical comparisons between inhibitor-based strategies, (ii) the absence of polymicrobial testing, and (iii) the challenge posed by AmpC-producing Enterobacterales. AmpC enzymes may mask ESBL activity or generate ambiguous phenotypic profiles. In our study, some AmpC-producing isolates displayed atypical color patterns, highlighting the need for cautious interpretation and, potentially, complementary diagnostic approaches. Prospective clinical validation studies are therefore warranted to confirm the clinical impact of this assay.

### Conclusion

This assay provides rapid functional detection of the two most clinically relevant β-lactam resistance mechanisms in Enterobacterales within a single workflow. The simplicity, low cost, and reagent stability of the proposed assay position the ESBL-Carba Combo PN test as a step toward more accessible and scalable phenotypic screening of major β-lactam resistance mechanisms in Enterobacterales. By combining ESBL and carbapenemase detection with rapid carbapenemase class discrimination, this assay provides accurate functional information that may facilitate earlier optimization of antimicrobial therapy and support antimicrobial stewardship initiatives.

## SUPPLEMENTAL MATERIAL

Supplemental Material spectrum.01123-26-s0001.docx Fig. S1 and S2; Tables S1 and S2.

ASM does not own the copyrights to Supplemental Material that may be linked to, or accessed through, an article. The authors have granted ASM a non-exclusive, world-wide license to publish the Supplemental Material files. Please contact the corresponding author directly for reuse.
