# Supplementary material for: Rapid ESBL-Carba Combo PN test for simultaneous detection of ESBL and carbapenemase activity in Enterobacterales
Source: Microbiol Spectr. 2026 Sep 2;14(10):e01123-26. doi: 10.1128/spectrum.01123-26 (PMC13689434; doi:10.1128/spectrum.01123-26)
Supplement: Supplementary file 1 [file spectrum.01123-26-s0001.docx]

**Supplementary Figure 1.** Schematic representation (A) and interpretation (B) of rapid detection of ESBL.

Rapid detection of ESBL (tubes A, B and C); _d_H_2_O: distilled water; CTX: cefotaxime; TAZ: tazobactam.

 **Supplementary Figure 2.** Schematic representation (A) and interpretation (B) of rapid detection of carbapenemase activity and classification.

Rapid detection of carbapenemases (tubes from A to E); _d_H_2_O: distilled water; ETP: ertapenem; DPA: dipicolinic acid; AVI: avibactam; REL: relebactam.

**Supplementary Table 1.** Results of the ESBL detection assay on Enterobacterales isolates after 20 minutes and 1 hour of incubation.

| **ID** | **Strain** | **Phenotype of β-lactam resistance** | **Genetic characterization** | | | **20 minutes of incubation** | | | **Interpretation** | **1 hour of incubation** | | | **Interpretation** |
| --- | --- | --- | --- | --- | --- | --- | --- | --- | --- | --- | --- | --- | --- |
|  |  |  | **ESBLs** | **Carbapenemases** | **Adquired β-lactamases** | **-** | **+ CTX** | **+CTX/**  **TAZ** |  | **-** | **+ CTX** | **+CTX/**  **TAZ** |  |
| 1 | *K. pneumoniae* | *ESBL* | CTX-M-2 | KPC-2 | OXA-9, TEM-1 | + | + | - | **✔** | + | + | - | **✔** |
| 2 | *E. coli* |  | CTX-M-3 | - | TEM-239 | + | + | - | **✔** | + | + | - | **✔** |
| 3 | *E. coli* |  | CTX-M-3 | - | TEM-224 | + | - | - | **FN** | + | + | - | **✔** |
| 4 | *E. coli* |  | CTX-M-9 | - | CMY-226 | + | + | - | **✔** | + | + | - | **✔** |
| 5 | *E. coli* |  | CTX-M-14 | - | TEM-1 | - | - | - | **FN** | - | - | - | **FN** |
| 6 | *E. coli* |  | CTX-M-15 | - | TEM-239 | + | + | - | **✔** | + | + | - | **✔** |
| 7 | *K. pneumoniae* |  | CTX-M-15 | - | OXA-1, TEM-1 | + | + | - | **✔** | + | + | - | **✔** |
| 8 | *E. cloacae* |  | CTX-M-15 | - | OXA-1, TEM-1 | + | + | - | **✔** | + | + | - | **✔** |
| 9 | *E. coli* |  | CTX-M-15 | - | CMY-16, TEM-1 | + | + | - | **✔** | + | + | - | **✔** |
| 10 | *E. coli* |  | CTX-M-15 | OXA-48 | TEM-1 | + | + | - | **✔** | + | + | - | **✔** |
| 11 | *E. coli* |  | CTX-M-14 | OXA-181 | - | + | + | - | **✔** | + | + | - | **✔** |
| 12 | *E. coli* |  | CTX-M-15 | OXA-181 | - | + | + | - | **✔** | + | + | - | **✔** |
| 13 | *E. coli* |  | CTX-M-15 | OXA-48 | OXA-1, TEM-1 | + | + | - | **✔** | + | + | - | **✔** |
| 14 | *E. coli* |  | CTX-M-15 | OXA-48 | OXA-1, TEM-1 | + | + | - | **✔** | + | + | - | **✔** |
| 15 | *E. coli* |  | CTX-M-15 | OXA-48 | OXA-1, TEM-1 | + | + | - | **✔** | + | + | - | **✔** |
| 16 | *K. pneumoniae* |  | CTX-M-15 | OXA-48 | - | + | + | - | **✔** | + | + | - | **✔** |
| 17 | *K. pneumoniae* |  | CTX-M-15 | OXA-48 | - | + | + | - | **✔** | + | + | - | **✔** |
| 18 | *E. coli* |  | CTX-M-15 | OXA-181 | - | + | + | - | **✔** | + | + | - | **✔** |
| 19 | *E. coli* |  | CTX-M-15 | OXA-181 | - | + | + | - | **✔** | + | + | - | **✔** |
| 20 | *E. coli* |  | CTX-M-15 | OXA-181 | - | + | + | - | **✔** | + | + | - | **✔** |
| 21 | *K. pneumoniae* |  | CTX-M-15 | OXA-181 and NDM-1 | - | + | + | + | **✔** | + | + | + | **✔** |
| 22 | *K. pneumoniae* |  | CTX-M-15 | NDM-1 | TEM-1 | + | + | + | **✔** | + | + | + | **✔** |
| 23 | *K. pneumoniae* |  | CTX-M-15 | KPC-2 | TEM-1 | + | + | - | **✔** | + | + | - | **✔** |
| 24 | *E. coli* |  | CTX-M-15 | - | - | + | + | - | **✔** | + | + | - | **✔** |
| 25 | *E. coli* |  | CTX-M-15 | - | - | + | + | - | **✔** | + | + | - | **✔** |
| 26 | *E. coli* |  | CTX-M-15 | - | - | + | + | - | **✔** | + | + | - | **✔** |
| 27 | *E. coli* |  | CTX-M-15 | - | - | + | + | - | **✔** | + | + | - | **✔** |
| 28 | *E. coli* |  | CTX-M-15 | - | - | + | + | - | **✔** | + | + | - | **✔** |
| 29 | *E. coli* |  | CTX-M-15 | - | - | + | + | - | **✔** | + | + | - | **✔** |
| 30 | *K. pneumoniae* |  | CTX-M-15 | - | - | + | + | - | **✔** | + | + | - | **✔** |
| 31 | *E. coli* |  | CTX-M-15 | - | CMY-42 | + | + | - | **✔** | + | + | - | **✔** |
| 32 | *E. coli* |  | CTX-M-15 | - | - | + | + | - | **✔** | + | + | - | **✔** |
| 33 | *E. coli* |  | CTX-M-15 | - | - | + | + | - | **✔** | + | + | - | **✔** |
| 34 | *K. pneumoniae* |  | CTX-M-33 | - | TEM-1, OXA-1 | + | + | - | **✔** | + | + | - | **✔** |
| 35 | *E. coli* |  | CTX-M-55 | - | TEM-239 | + | + | - | **✔** | + | + | - | **✔** |
| 36 | *E. coli* |  | SHV-12 | - | TEM-1 | + | - | - | **FN** | + | + | - | **✔** |
| 37 | *E. coli* |  | SHV-12 | - | - | + | + | - | **✔** | + | + | - | **✔** |
| 38 | *K. pneumoniae* |  | SHV-12 | - | - | + | + | - | **✔** | + | + | - | **✔** |
| 39 | *K. pneumoniae* |  | - | OXA-48 | - | + | + | - | **✔** | + | + | - | **✔** |
| 40 | *E. coli* |  | - | OXA-181 | - | + | + | - | **✔** | + | + | - | **✔** |
| 41 | *K. pneumoniae* |  | GES-1 | - | - | + | + | - | **✔** | + | + | - | **✔** |
| 42 | *E. cloacae* |  | PER-1 | - | - | + | + | - | **✔** | + | + | - | **✔** |
| 43 | *E. coli* |  | VEB-1 | - | OXA-10, TEM-1 | + | + | - | **✔** | + | + | - | **✔** |
| 44 | *K. pneumoniae* |  | OXA-2 | - | - | + | + | - | **✔** | + | + | - | **✔** |
| 45 | *E. coli* | *Penicillinase* | - | - | TEM-239 | + | - | - | **✔** | + | - | - | **✔** |
| 46 | *E. coli* |  | - | - | TEM-1, CMY-2 | + | - | - | **✔** | + | - | - | **✔** |
| 47 | *K. pneumoniae* |  | - | - | - | + | - | - | **✔** | + | - | - | **✔** |
| 48 | *K. pneumoniae* |  | - | - | - | + | - | - | **✔** | + | - | - | **✔** |
| 49 | *E. coli* | *Carbapenemase producers* | - | KPC-2 | TEM-1, OXA-1 | + | + | - | **✔** | + | + | - | **✔** |
| 50 | *K. pneumoniae* |  | - | KPC-2 | - | + | + | - | **✔** | + | + | - | **✔** |
| 51 | *K. pneumoniae* |  | - | KPC-3 | - | + | + | - | **✔** | + | + | - | **✔** |
| 52 | *E. cloacae* |  | - | GES-5 | - | + | + | - | **✔** | + | + | - | **✔** |
| 53 | *K. pneumoniae* |  | - | IMP-1 | - | + | + | + | **✔** | + | + | + | **✔** |
| 54 | *K. pneumoniae* |  | OXA-2 | IMP-1 | - | + | + | + | **✔** | + | + | + | **✔** |
| 55 | *E. coli* |  | - | NDM-1 | - | + | + | + | **✔** | + | + | + | **✔** |
| 56 | *E. coli* |  | - | NDM-1 | - | + | + | + | **✔** | + | + | + | **✔** |
| 57 | *E. coli* |  | - | NDM-1 | - | + | + | + | **✔** | + | + | + | **✔** |
| 58 | *E. coli* |  | - | NDM-1 | - | + | + | + | **✔** | + | + | + | **✔** |
| 59 | *E. coli* |  | - | NDM-1 | - | + | + | + | **✔** | + | + | + | **✔** |
| 60 | *E. cloacae* |  | - | NDM-4 | - | + | + | + | **✔** | + | + | + | **✔** |
| 61 | *E. coli* |  | - | NDM-4 | - | + | + | + | **✔** | + | + | + | **✔** |
| 62 | *E. coli* |  | - | NDM-5 | - | + | + | + | **✔** | + | + | + | **✔** |
| 63 | *E. coli* |  | - | NDM-5 | - | + | + | + | **✔** | + | + | + | **✔** |
| 64 | *E. cloacae* | *No resistance* | - | - | - | + | - | - | **✔** | + | - | - | **✔** |
| 65 | *E. coli* |  | - | - | - | - | - | - | **✔** | - | - | - | **✔** |
| 66 | *E. coli* |  | - | - | - | - | - | - | **✔** | - | - | - | **✔** |
| 67 | *E. coli* |  | *-* | *-* | - | - | - | - | **✔** | - | - | - | **✔** |

✔️: Expected result; FN: False negative.

**Supplementary Table 2.** Results of the carbapenemase detection and classification assay on Enterobacterales isolates after 20 minutes and 1 hour of incubation.

| ID | Strain | Ambler class | Genetic characterization | | | 20 minutes of incubation | | | | | Interpretation | 1 hour of incubation | | | | | Interpretation |
| --- | --- | --- | --- | --- | --- | --- | --- | --- | --- | --- | --- | --- | --- | --- | --- | --- | --- |
|  |  |  | Carbapenemases | ESBL | Adquired β-lactamases | - | + ETP | +ETP/DPA | +ETP/AVI | +ETP/REL |  | - | + ETP | +ETP/DPA | +ETP/AVI | +ETP/REL |  |
| 1 | *E. cloacae* | *CLASS A* | KPC-2 | - | OXA-1 | + | + | + | - | - | **✔** | + | + | + | - | - | **✔** |
| 2 | *K. pneumoniae* |  | KPC-2 | CTX-M-15 | OXA-1, TEM-1 | + | + | + | - | - | **✔** | + | + | + | - | - | **✔** |
| 3 | *K. pneumoniae* |  | KPC-2 | - | TEM-1 | + | + | + | - | - | **✔** | + | + | + | - | - | **✔** |
| 4 | *K. pneumoniae* |  | KPC-2 | - | TEM-1 | + | + | + | - | - | **✔** | + | + | + | - | - | **✔** |
| 5 | *K. pneumoniae* |  | KPC-2 | CTX-M-15 | OXA-1, TEM-1 | + | + | + | - | - | **✔** | + | + | + | - | - | **✔** |
| 6 | *K. pneumoniae* |  | KPC-3 | - | - | + | + | + | - | - | **✔** | + | + | + | - | - | **✔** |
| 7 | *K. pneumoniae* |  | KPC-3 | SHV-11 | - | + | + | + | - | - | **✔** | + | + | + | - | - | **✔** |
| 8 | *K. pneumoniae* |  | KPC-3 | - | TEM-1 | + | + | + | - | - | **✔** | + | + | + | - | - | **✔** |
| 9 | *K. pneumoniae* |  | KPC-3 | SHV-11 | CMY-16 | + | + | + | - | - | **✔** | + | + | + | - | - | **✔** |
| 10 | *K. pneumoniae* |  | KPC-3 | CTX-M-15 | CTX-M-15, OXA-1 | + | - | + | - | - | **✔** | + | + | + | - | - | **✔** |
| 11 | *K. pneumoniae* |  | KPC-3 | - | TEM-1, OXA-9 | + | + | + | - | - | **✔** | + | + | + | - | - | **✔** |
| 12 | *K. pneumoniae* |  | KPC-3 | - | TEM-1 | + | + | + | - | - | **✔** | + | + | + | - | - | **✔** |
| 13 | *K. pneumoniae* |  | KPC-3 | CTX-M-15 | - | + | + | + | - | - | **✔** | + | + | + | - | - | **✔** |
| 14 | *K. pneumoniae* |  | KPC-3 | CTX-M-15 | - | + | + | + | - | - | **✔** | + | + | + | - | - | **✔** |
| 15 | *E. cloacae* | *CLASS B* | NDM-1 | - | OXA-1 | + | + | - | + | + | **✔** | + | + | - | + | + | **✔** |
| 16 | *E. coli* |  | NDM-1 | - | OXA-1, CMY-4 | + | + | - | + | + | **✔** | + | + | - | + | + | **✔** |
| 17 | *K. pneumoniae* |  | NDM-1 | - | TEM-1 | + | + | - | + | + | **✔** | + | + | - | + | + | **✔** |
| 18 | *K. pneumoniae* |  | NDM-1 | - | - | + | + | - | + | + | **✔** | + | + | - | + | + | **✔** |
| 19 | *K. pneumoniae* |  | NDM-1 | CTX-M-15 | CMY-4, TEM-1 | + | + | - | + | + | **✔** | + | + | - | + | + | **✔** |
| 20 | *K. pneumoniae* |  | NDM-1 | CTX-M-15 | OXA-1, TEM-1, OXA-9 | + | + | - | + | + | **✔** | + | + | - | + | + | **✔** |
| 21 | *K. pneumoniae* |  | NDM-1 | CTX-M-15 | LAP-2 | + | + | - | + | + | **✔** | + | + | - | + | + | **✔** |
| 22 | *K. pneumoniae* |  | NDM-1 | CTX-M-15 | LAP-2 | + | + | - | + | + | **✔** | + | + | - | + | + | **✔** |
| 23 | *K. pneumoniae* |  | NDM-1 | CTX-M-15 | - | + | + | - | + | + | **✔** | + | + | - | + | + | **✔** |
| 24 | *K. pneumoniae* |  | NDM-1 | CTX-M-15 | OXA-1, TEM-1, OXA-9 | + | + | - | + | + | **✔** | + | + | - | + | + | **✔** |
| 25 | *K. pneumoniae* |  | NDM-1 | CTX-M-15 | TEM-1 | + | + | - | + | + | **✔** | + | + | - | + | + | **✔** |
| 26 | *E. coli* |  | NDM-5 | - | - | + | + | - | + | + | **✔** | + | + | - | + | + | **✔** |
| 27 | *E. coli* |  | NDM-5 | - | - | + | + | - | + | + | **✔** | + | + | - | + | + | **✔** |
| 28 | *E. coli* |  | NDM-5 | - | CMY-42 | + | + | - | + | + | **✔** | + | + | - | + | + | **✔** |
| 29 | *E. coli* |  | NDM-5 | CTX-M-15 | CMY-42, TEM-1 | + | + | - | + | + | **✔** | + | + | - | + | + | **✔** |
| 30 | *E. coli* |  | NDM-5 | - | CMY-42, TEM-1 | + | + | - | + | + | **✔** | + | + | - | + | + | **✔** |
| 31 | *E. coli* |  | NDM-5 | - | OXA-10 | + | + | - | + | + | **✔** | + | + | - | + | + | **✔** |
| 32 | *E. coli* |  | NDM-5 | CTX-M-15 | OXA-1 | + | + | - | + | + | **✔** | + | + | - | + | + | **✔** |
| 33 | *K. pneumoniae* |  | NDM-7 | - | - | + | + | - | + | + | **✔** | + | + | - | + | + | **✔** |
| 34 | *E. coli* |  | NDM-19 | - | - | + | + | - | + | + | **✔** | + | + | - | + | + | **✔** |
| 35 | *E. cloacae* |  | VIM-1 | - | - | + | + | - | + | + | **✔** | + | + | - | + | + | **✔** |
| 36 | *K. pneumoniae* |  | VIM-1 | SHV-5 | - | + | + | - | + | + | **✔** | + | + | - | + | + | **✔** |
| 37 | *K. pneumoniae* |  | VIM-1 | - | - | + | + | - | + | + | **✔** | + | + | - | + | + | **✔** |
| 38 | *K. pneumoniae* |  | VIM-1 | - | - | + | + | - | + | + | **✔** | + | + | - | + | + | **✔** |
| 39 | *E. cloacae* |  | VIM-4 | - | - | + | + | - | + | + | **✔** | + | + | - | + | + | **✔** |
| 40 | *K. pneumoniae* |  | VIM-4 | - | - | + | + | - | + | + | **✔** | + | + | - | + | + | **✔** |
| 41 | *E. coli* |  | VIM-19 | - | CMY-4 | + | + | - | + | + | **✔** | + | + | - | + | + | **✔** |
| 42 | *K. pneumoniae* |  | VIM-19 | CTX-M-3, TEM-10 | CMY-4 | + | + | - | + | + | **✔** | + | + | - | + | + | **✔** |
| 43 | *K. pneumoniae* |  | VIM-19 | CTX-M-3 | TEM-1 | + | + | - | + | + | **✔** | + | + | - | + | + | **✔** |
| 44 | *K. pneumoniae* |  | IMP-1 | TEM-15 | - | + | + | - | + | + | **✔** | + | + | - | + | + | **✔** |
| 45 | *K. pneumoniae* |  | IMP-1 | TEM-15, OXA-2 | - | + | + | - | + | + | **✔** | + | + | - | + | + | **✔** |
| 46 | *K. pneumoniae* |  | IMP-1 | TEM-15, OXA-2 | - | + | + | - | + | + | **✔** | + | + | - | + | + | **✔** |
| 47 | *K. pneumoniae* |  | IMP-4 | - | TEM-1 | + | + | - | + | + | **✔** | + | + | - | + | + | **✔** |
| 48 | *K. pneumoniae* |  | IMP-8 | - | LAP-2 | + | + | - | + | + | **✔** | + | + | - | + | + | **✔** |
| 49 | *P. mirabilis* | *CLASS D* | OXA-23 | - | - | + | + | + | - | + | **✔** | + | + | + | - | + | **✔** |
| 50 | *E. coli* |  | OXA-48 | - | - | + | + | + | - | + | **✔** | + | + | + | - | + | **✔** |
| 51 | *E. cloacae* |  | OXA-48 | CTX-M-9 | - | + | + | + | - | + | **✔** | + | + | + | - | + | **✔** |
| 52 | *E. coli* |  | OXA-48 | CTX-M-14 | - | + | + | + | - | + | **✔** | + | + | + | - | + | **✔** |
| 53 | *E. coli* |  | OXA-48 | CTX-M-24 | - | + | + | + | - | + | **✔** | + | + | + | - | + | **✔** |
| 54 | *E. coli* |  | OXA-48 |  | - | + | + | + | - | + | **✔** | + | + | + | - | + | **✔** |
| 55 | *E. coli* |  | OXA-48 | CTX-M-14 | - | + | + | + | - | + | **✔** | + | + | + | - | + | **✔** |
| 56 | *K. pneumoniae* |  | OXA-48 | CTX-M-15 | TEM-1, OXA-1 | + | + | + | - | + | **✔** | + | + | + | - | + | **✔** |
| 57 | *K. pneumoniae* |  | OXA-244 | SHV-11 | - | + | + | + | - | + | **✔** | + | + | + | - | + | **✔** |
| 58 | *K. pneumoniae* |  | OXA-48 | CTX-M-15 | OXA-1 | + | + | + | - | + | **✔** | + | + | + | - | + | **✔** |
| 59 | *K. pneumoniae* |  | OXA-48 | CTX-M-15 | - | + | + | + | - | + | **✔** | + | + | + | - | + | **✔** |
| 60 | *K. pneumoniae* |  | OXA-48 | CTX-M-15 | OXA-1 | + | + | + | - | + | **✔** | + | + | + | - | + | **✔** |
| 61 | *K. pneumoniae* |  | OXA-48 | CTX-M-15 | OXA-1 | + | + | + | - | + | **✔** | + | + | + | - | + | **✔** |
| 62 | *K. pneumoniae* |  | OXA-204 | CTX-M-14 | OXA-1, TEM-1, CMY-4 | + | + | + | - | - | **M** | + | + | + | - | + | **✔** |
| 63 | *E. coli* |  | OXA-204 | CTX-M-15 | OXA-1 | + | + | + | - | + | **✔** | + | + | + | - | + | **✔** |
| 64 | *K. pneumoniae* |  | OXA-232 | CTX-M-15 | OXA-1 | + | + | + | - | + | **✔** | + | + | + | - | + | **✔** |
| 65 | *E. coli* |  | OXA-244 | - | - | + | + | + | - | + | **✔** | + | + | + | - | + | **✔** |
| 66 | *E. coli* |  | OXA-244 | - | - | + | + | + | - | + | **✔** | + | + | + | - | + | **✔** |
| 67 | *E. coli* |  | OXA-244 | - | - | + | + | + | - | + | **✔** | + | + | + | - | + | **✔** |
| 68 | *E. coli* |  | OXA-244 | CTX-M-14 | TEM-1 | + | + | + | - | - | **M** | + | + | + | - | - | **M** |
| 69 | *E. coli* |  | OXA-244 | - | - | + | + | + | - | + | **✔** | + | + | + | - | + | **✔** |
| 70 | *E. coli* |  | OXA-244 | CTX-M-27 | - | + | + | + | - | + | **✔** | + | + | + | - | + | **✔** |
| 71 | *E. coli* |  | OXA-244 | - | - | + | + | + | - | + | **✔** | + | + | + | - | + | **✔** |
| 72 | *E. coli* |  | OXA-244 | CTX-M-14 | TEM-1 | + | + | + | - | + | **✔** | + | + | + | - | + | **✔** |
| 73 | *E. coli* |  | OXA-244 | CTX-M-27 | - | + | + | + | - | + | **✔** | + | + | + | - | + | **✔** |
| 74 | *E. coli* |  | OXA-244 | CTX-M-14 | TEM-1 | + | + | + | - | + | **✔** | + | + | + | - | + | **✔** |
| 75 | *E. coli* | *Double carbapenemases* | NDM-5 and OXA-244 |  | - | + | + | + | + | + | **✔** | + | + | + | + | + | **✔** |
| 76 | *K. pneumoniae* | *Non-carbapenemase producers* | - | CTX-M-3 | TEM-1 | + | - | - | - | - | **✔** | + | - | - | - | - | **✔** |
| 77 | *E. coli* |  | - | CTX-M-3 | - | + | - | - | - | - | **✔** | + | - | - | - | - | **✔** |
| 78 | *K. pneumoniae* |  | - | CTX-M-15 | - | + | - | - | - | - | **✔** | + | - | - | - | - | **✔** |
| 79 | *K. pneumoniae* |  | - | CTX-M-15 | - | + | - | - | - | - | **✔** | + | - | - | - | - | **✔** |
| 80 | *K. pneumoniae* |  | - | CTX-M 15 | - | + | - | - | - | - | **✔** | + | - | - | - | - | **✔** |
| 81 | *E. cloacae* |  | - | CTX-M-3 | TEM-1 | + | - | - | - | - | **✔** | + | - | - | - | - | **✔** |
| 82 | *E. coli* |  | - | - | TEM-1 | + | - | - | - | - | **✔** | + | - | - | - | - | **✔** |
| 83 | *E. cloacae* |  | - | - | TEM-3 | + | - | - | - | - | **✔** | + | - | - | - | - | **✔** |
| 84 | *E. coli* |  | - | - | OXA-1 | + | - | - | - | - | **✔** | + | - | - | - | - | **✔** |
| 85 | *K. pneumoniae* |  | - | OXA-163 | - | + | - | - | - | - | **✔** | + | - | - | - | - | **✔** |
| 86 | *K. pneumoniae* |  | - | - | - | + | - | - | - | - | **✔** | + | - | - | - | - | **✔** |
| 87 | *K. pneumoniae* |  | - | - | - | + | - | - | - | - | **✔** | + | - | - | - | - | **✔** |
| 88 | *K. pneumoniae* |  | - | - | - | + | - | - | - | - | **✔** | + | - | - | - | - | **✔** |
| 89 | *K. pneumoniae* |  | - | - | DHA-1 | + | - | - | - | - | **✔** | + | - | - | - | - | **✔** |
| 90 | *E. coli* |  | - | - | - | - | - | - | - | - | **✔** | - | - | - | - | - | **✔** |
| 91 | *E. coli* |  | - | - | - | - | - | - | - | - | **✔** | - | - | - | - | - | **✔** |
| 92 | *E. coli* |  | *-* | *-* | - | - | - | - | - | - | **✔** | - | - | - | - | - | **✔** |

✔️: Expected result; M: Misidentification of carbapenemase class.
